# Chemical Composition and *In Vitro* Antibacterial Activity of *Mentha spicata* Essential Oil against Common Food-Borne Pathogenic Bacteria

**DOI:** 10.1155/2015/916305

**Published:** 2015-08-16

**Authors:** Yasser Shahbazi

**Affiliations:** Department of Food Hygiene and Quality Control, Faculty of Veterinary Medicine, Razi University, Kermanshah, Iran

## Abstract

The aim of the present study was to investigate chemical composition and antibacterial activity of essential oil from the leaf of *Mentha spicata* plant against common food-borne pathogenic bacteria (*Staphylococcus aureus, Bacillus subtilis, Bacillus cereus, Listeria monocytogenes, Salmonella typhimurium*, and *Escherichia coli* O157:H7). Chemical composition of the essential oil was identified by gas chromatography coupled with mass spectrometer detector (GC-MS). The antibacterial activity of the essential oil was evaluated by broth microdilution method and agar disk diffusion assay. According to the result of GC-MS analysis, 18 components were identified, accounting for 99.89% of the whole essential oil. The main components were carvone (78.76%), limonene (11.50%), *β*-bourbonene (11.23%), *cis*-dihydrocarveol (1.43%), *trans*-caryophyllene (1.04%), menthone (1.01%), menthol (1%), and terpinen-4-ol (0.99). The essential oil exhibited moderate level of antibacterial activity against all test microorganisms. In general, Gram-positive bacteria were more susceptible to *M. spicata* essential oil than Gram-negative bacteria. *L. monocytogenes* was the most sensitive of the microorganisms to the antibacterial activity of *M. spicata* essential oil (inhibition zone = 22 mm and MIC and MBC = 2.5 *µ*L/mL). Based on our results, the essential oil of *M. spicata* plant collected from Kermanshah province, west of Iran, has a potential to be applied as antibacterial agent.

## 1. Introduction

Due to increasing consumers' concerns regarding processed and ready-to-eat foods containing antibiotics, pesticides, hormones, and synthetic additives and also increasing demand to replace artificial antimicrobial agents with natural alternatives, the usage of natural and organic foods has been experiencing explosive market growth [1–3]. However, the untreated products and natural foods may be more susceptible to growth of food-borne pathogens than the conventional food version [4]. The most important food-borne pathogenic bacteria that have survived and grow in these products include* Staphylococcus aureus*,* Bacillus* spp.,* Listeria monocytogenes*,* Salmonella* spp.* Escherichia coli*,* Yersinia* spp., and* Clostridium* spp. [5, 6]. These bacteria cause a great proportion of food-borne outbreaks in different foods such as dairy products, vegetables, and meat and fish products [7]. In this context, plant essential oils are attracting interest as natural food preservatives in order to ensure the safety of food [8].

Essential oils are volatile aromatic components that are obtained from different parts of plants including bud, seed, root, leave, stem, wood, bark, and flower [8, 9]. Generally, essential oils have been widely used in the preparation of cosmetics, drugs, and perfumery [10]. The genus* Mentha*, one of the most important members of the Lamiaceae family, is represented by 19 species and 13 natural hybrids. The most common and popular mint for cultivation is* M. spicata* [11]. It is widely grown in temperate areas of the world, particularly Africa, Temperate Asia, and Europe, but nowadays it was cultivated throughout all regions of the world [12]. In Iran, the fresh and dried plants and their essential oils have long been used as a flavoring agent in various food products, including cheese and doogh (Iranian yoghurt drink), chocolate, beverages, jellies, syrups, candies, ice creams, and chewing gum [13, 14]. Moreover, it has been extensively applied to treatment of various diseases such as nausea, vomiting, and gastrointestinal disorders and also as breath freshener, antiseptic mouth rinse, and toothpaste [11, 15]. The main constituents of the* M. spicata* essential oil are phenolic compounds such as carvone and limonene [16].

Based on our Knowledge, in comparison to various aromatic plant species such as garlic, thyme, and clove, there is little information about chemical composition and antimicrobial activity of the* M. spicata* essential oil collected from west part of Iran. Hence, the aims of the present study were to (1) determinate chemical composition of* M. spicata* essential oil collected from Gilane Gharb city, Kermanshah province (west of Iran), by GC-MS, and (2) investigate antimicrobial activity of the essential oil against common food-borne bacteria (*Staphylococcus aureus*,* Bacillus subtilis*,* Bacillus cereus*,* Listeria monocytogenes*,* Salmonella typhimurium*, and* Escherichia coli* O157:H7) by broth microdilution method and agar disk diffusion assay.

## 2. Materials and Methods

### 2.1. Collection of Plant Material

The fresh leaves of* M. spicata* were obtained from Gilane Gharb city, Kermanshah province, west of Iran (latitude: 3,776,583 Universal Transverse Mercator (UTM), longitude: 585,86 UTM, and altitude: 833 m above sea level (a.s.l.)), during the full-blooming stage in March–July 2014. Botanical identification of the plant was done by Dr. Seyed Mohammad Masoumi (Faculty of Agriculture, Razi University, Kermanshah, Iran) and a representative voucher specimen (number 1267) has been placed in the Herbarium of the Research Center of Natural Resources of Tehran, Iran.

### 2.2. Essential Oil Isolation

The air-dried leaf (100 g) of the* M. spicata* plant was hydrodistilled for 3.5 h by using clevenger-type apparatus according to standard technique [17]. The collected oil layer on top of the aqueous distillate was separated, dried over anhydrous sodium sulfate (0.5 g) (Merck, Darmstadt, Germany), filtered through a 0.22 *μ*m filter (Millipore, Bedford MA, USA), kept in sealed airtight glass vials, and stored at 4°C prior to further analysis (chemical analysis and antibacterial tests).

### 2.3. Gas Chromatography-Mass Spectrometry (GC-MS) Analysis of Essential Oil

The most volatile compounds of* M. spicata* essential oil were identified and quantified by means of analytical gas chromatography coupled with mass spectrometer detector (GC-MS) (Thermo Quest Finningan, UK). The GC-MS apparatus was equipped with HP-5MS 5% phenyl methyl silicone capillary column (30.00 m length × 0.25 mm ID and 0.25 *μ*m film thickness). The temperature of mass transfer line and injector was set at 290°C and 250°C, respectively. The initial oven temperature was kept at 50°C, raised to 120°C at program ramp rate 2.5°C per min, and then raised to 265°C and kept for 6 min. Helium with certified purity greater than 99% was used as carrier gas, at constant flow rate of 1.2 mL/min. 1 *μ*L of the sample was injected automatically in the split mode (split ratio 1 : 20). The GC-MS analysis was conducted in triplicate. The mass spectra were obtained by electron ionization (EI) at 70 eV. Moreover, the chemical analysis of the essential oil was carried out by analytical gas chromatography (GC) (Thermo Quest Finningan, UK) with the same temperature program as described above. The GC analysis was done in triplicate. Identification of the major constituents of the essential oil was accompanied based on comparison between their retention indices (RIs) with retention indices of published data. Further identification was made by matching their recorded mass spectra with those stored in Wiley/NBS mass spectral library of the GC-MS data system. The percentage of each essential oil composition was calculated from GC peak areas without using correction factors.

### 2.4. Preparation of Test Microorganisms

The antibacterial activity of* M. spicata* essential oil was tested against bacteria including* S. aureus* (ATCC 6538, ATCC 29213, and ATCC 25923),* B. subtilis* (ATCC 6633),* B. cereus* (ATCC 11774),* L. monocytogenes* (ATCC 19118),* S. typhimurium* (ATCC 14028), and* E. coli* O157:H7 (ATCC 10536 and ATCC 25922). All strains were purchased as lyophilized cultures from the culture collection of the Iranian Research Organization for Science and Technology (IROST), Tehran, Iran. The bacterial cultures were maintained at 4°C on Brain Heart Infusion agar (BHI, Merck, Darmstadt, Germany) and were reactivated by subculture twice before testing. For the preparation microbial inoculants of bacteria (1 × 10^6^ CFU/mL), three to five well-isolated colonies were removed with a sterile wire loop, inoculated into a tube containing 5 mL of BHI broth medium (Merck, Darmstadt, Germany), and incubated at 37°C for 18 h. After 18 h, the suspensions were adjusted to 0.5 McFarland standard turbidity and diluted to the appropriate concentration. Also, the number of bacterial colonies was enumerated by triplicate plating on BHI agar medium.

### 2.5. Antibacterial Tests

#### 2.5.1. Determination of the Minimum Inhibitory Concentration (MIC) and the Minimum Bactericidal Concentration (MBC)

The broth microdilution method was used to determine the minimum inhibitory concentration (MIC) and the minimum bactericidal concentration (MBC) according to guideline of the National Committee for Clinical Laboratory Standards [18]. Firstly, 5% (v/v) dimethyl sulfoxide (DMSO) (Merck, Darmstadt, Germany) as an emulsifier and 0.05% (w/v) agar-agar (Merck, Darmstadt, Germany) as a stabilizer of the essential oil were added in 10 mL Mueller-Hinton Broth (MHB, Merck, Darmstadt, Germany) medium. After autoclaving of medium, different concentrations of the essential oil (0.017, 0.035, 0.07, 0.156, 0.312, 0.625, 1.25, 5, 10, 20, and 40 *μ*L/mL) were set up in MHB medium. The 96-well plastic microdilution tray with round bottom wells (Extragene, USA) were prepared by dispensing into each well 90 *μ*L of MHB containing different concentrations of the essential oil and 10 *μ*L of the bacterial inoculum, which was approximately 10^6^ CFU/mL. Positive and negative controls were considered as follows: positive control: MHB containing DMSO and bacterial inoculum without essential oil and negative control: MHB containing DMSO and essential oil without bacterial inoculum. Contents of each well were mixed on a plate shaker at 250 rpm for 20 s and incubated at 37°C for 18 h. The lowest concentration of the essential oil showing visually no growth (by comparing with the first growth control) was taken as MIC. Determination of the minimum concentration of the essential oil that reduces 99.99% of population bacteria (MBC) was done by culturing of 10 *μ*L of each well without any invisible growth. The culturing was performed on Mueller-Hinton agar (MHA, Merck, Darmstadt, Germany) medium and incubated at 37°C for 24 h.* S. aureus* (ATCC 29213) was considered the control microorganism for broth microdilution method.

#### 2.5.2. Agar Disk Diffusion Assay

The antibacterial activity of* M. spicata* essential oil was also examined by agar disk diffusion assay according to guideline of the National Committee for Clinical Laboratory Standards [19]. Within 15 min, after adjusting the turbidity of the inoculum suspensions, a sterile cotton swab was dipped into the adjusted suspension and then spread on MHA medium by surface method. After 5 min, the filter paper disc (diameter 6 mm; Whatman number 1) impregnated with 20 *μ*L of the essential oil was placed on the surface of MHA medium. The plates were incubated 37°C overnight and examined for the zone of inhibition. The diameter of the inhibition zone was measured in millimeters, using sliding calipers. Positive (tetracycline) and negative (only DMSO) controls were considered in the present test.* S. aureus* (ATCC 25923) and* E. coli* (ATCC 25922) were considered the control organisms for disk diffusion. All Experiments were repeated in triplicate.

## 3. Results and Discussion

### 3.1. Chemical Composition of* M. spicata* Essential Oil

The result of GC-MS investigation of the* M. spicata* essential oil is shown in Table 1. According to our results, 18 components were identified by GC-MS, accounting for 99.89% of the whole essential oil. The main components were carvone (78.76%), limonene (11.50%), *β*-bourbonene (11.23%),* cis*-dihydrocarveol (1.43%),* trans*-caryophyllene (1.04%), menthone (1.01%), menthol (1%), and terpinen-4-ol (0.99). Our results about most abundant chemical compounds of* M. spicata* essential oil collected from west part of Iran are in accordance with previous studies [20–24]. Chauhan et al. (2009) reported carvones as the main components of the essential oil collected from India. Other constituents of their essential oil were limonene, 1,8-cineole,* Z-β*-ocimene, and* cis*-muurola-4 [20]. Sertkaya et al. (2010) and Mahboubi and Haghi (2008) reported carvones as the major constituents of the* M. spicata* essential oil [13, 21]. Moreover, Govindarajan et al. (2012) investigated chemical composition of* M. spicata* obtained from Tamilnadu, India, and reported carvone (48.60%),* cis*-carveol (21.30%), and limonene (11.30%) were the major components [22]. In contrast with our results, Telci et al. (2010) investigated antibacterial activity and chemical composition environmental variation of* M. spicata* and indicated that pulegone and piperitone were the major components [16]. Generally, the observed differences in chemical composition content of the essential oil, when compared with previous studies, could be attributed to several factors including different method used for extraction of the essential oil, geographical conditions, climate and seasonal variations, and the species and stage of the plant growth and processing of plant materials before extraction of the oil [1, 2, 8, 9, 13].

### 3.2. Antibacterial Activity

The antibacterial activity of the essential oil of* M. spicata* against common food-borne bacteria obtained by the broth microdilution test and agar disk diffusion assay was shown in Tables 2 and 3. The results of the current research revealed that the essential oil exhibited moderate antibacterial effect against the microorganisms. Generally, the results of the present study showed that Gram-positive bacteria were more susceptible to* M. spicata* essential oil than Gram-negative bacteria. The low susceptibility of Gram-negative bacteria could be attributed to the presence of hydrophobic lipopolysaccharide in their outer membrane which provides protection against different agents [3, 25, 26]. As shown in Table 3,* L. monocytogenes* was the most sensitive of the microorganisms to the antibacterial activity of* M. spicata* essential oil (inhibition zone = 22 mm).

Previous antibacterial studies showed that* M. spicata* essential oil had antibacterial effect on the growth of Gram-negative and Gram-positive bacteria. Mahboubi and Haghi (2008) examined the antibacterial effect of* M. spicata* essential oil by broth microdilution and disk diffusion methods and reported that the essential oil had high antibacterial activity against* S. aureus*,* L. monocytogenes*,* B. cereus*, and* E. coli* [13]. These researchers reported that MIC values of essential oil against* S. aureus, L. monocytogenes, B. cereus,* and* E. coli* were 0.5, 1, 1, and 4 *μ*L/mL, respectively. Different reported MICs in this study may be the result of using different bacterial species and media and amount of phenolic compounds of the essential oil. Moreover, Gulluce et al. (2007) indicated the essential oil of* M. longifolia* collected from Turkey had strong antibacterial activity against numerous bacteria such as* S. aureus*,* B. subtilis*,* B. cereus*,* L. monocytogenes*,* S. typhimurium,* and* E. coli* O157:H7 [27]. Generally, it is believed that the antibacterial activities of essential oils are likely related to the percentage of phenols, terpenes, and aldoketones [27–29]. The antibacterial activity of* M. spicata* essential oil could be attributed to the presence of carvone and limonene. It has been reported that carvone is one of the most efficient antimicrobial agents of various plants [30]. The mechanism of antibacterial activity of carvone is not completely understood in great detail. It has been demonstrated that the mechanism of action of carvone on the growth microorganisms includes the destabilization of the phospholipid bilayer structure, interaction with membrane enzymes and proteins, and its act as a proton exchanger reducing the pH gradient across the membrane [31]. However, scanning electron microscope study is necessary to examine the accurate mechanism of action of* M. spicata* essential oil.

## 4. Conclusions

The results of the present study indicate that* M. spicata* essential oil has remarkable antibacterial activity against common food-borne bacteria associated with outbreaks (*S. aureus*,* B. subtilis*,* B. cereus*,* L. monocytogenes*,* S. typhimurium*, and* E. coli* O157:H7). The antibacterial activity of the essential oil could be attributed to the presence of various active compounds. The main components were carvone (78.76%), limonene (11.50%), *β*-bourbonene (11.23%),* cis*-dihydrocarveol (1.43%),* trans*-caryophyllene (1.04%), menthone (1.01%), menthol (1%), and terpinen-4-ol (0.99). Based on our results, the essential oil of* Mentha* plant collected from Kermanshah province, west of Iran, has a potential to be applied as antibacterial agent.

## Figures and Tables

**Table 1:** Essential oil composition of *M. spicata* identified by GC-MS.

Number	Compound	Retention index	Percentage
1	*β*-Myrcene	450	0.25
2	Limonene	509	11.50
3	*γ*-Terpinene	553	0.16
4	Menthone	703	1.01
5	Menthol	713	1
6	Terpinen-4-ol	720	0.99
7	*α*-Terpinol	737	0.31
8	Dihydrocarveol	742	0.22
9	*cis*-Dihydrocarveol	746	1.43
10	Dihydrocarvone	756	0.43
11	*trans*-Carveol	773	0.3
12	Carvone	819	78.76
13	Dihydrocarvyl acetate	906	0.57
14	L-carveol	946	0.32
15	*β*-Bourbonene	981	1.23
16	*trans*-Caryophyllene	1021	1.04
17	*γ*-Amorphene	1048	0.21
18	*α*-Amorphene	1058	0.16
	Others	—	0.11
	Sum		100

**Table 2:** Antibacterial activity of *M. spicata* essential oil indicated as MIC/MBC.

Bacteria	MIC (*μ*L/mL)	MBC (*μ*L/mL)
*S. aureus* (ATCC 6538)	10	10
*S. aureus* (ATCC 29213)	8	8
*B. subtilis* (ATCC 6633)	2.5	5
*B. cereus* (ATCC 11774)	2.5	5
*L. monocytogenes* (ATCC 19118)	2.5	2.5
*S. typhimurium* (ATCC 14028)	10	10
*E. coli* O157:H7 (ATCC 10536)	10	10

**Table 3:** Antibacterial effect of *M. spicata* essential oil by agar disk diffusion assay.

Bacteria	Inhibition zone (mm)	
	Essential oil	Tetracycline
*S. aureus* (ATCC 6538)	10	26
*S. aureus* (ATCC 25923)	8	24
*B. subtilis* (ATCC 6633)	18	7
*B. cereus* (ATCC 11774)	14	34
*L. monocytogenes* (ATCC 19118)	22	23
*S. typhimurium* (ATCC 14028)	10	30
*E. coli* O157:H7 (ATCC 10536)	12	26
*E. coli* (ATCC 25922)	10	25
